# Neurostimulation and Sense of Agency: Three tDCS Experiments on the Modulation of Intentional Binding

**DOI:** 10.3390/brainsci15020176

**Published:** 2025-02-11

**Authors:** Marika Bonuomo, Davide Perrotta, Gloria Di Filippo, Rinaldo Livio Perri

**Affiliations:** Department of Economical, Communication and Psychological Sciences, University Niccolò Cusano, 00166 Rome, Italy; marika.bonuomo@unicusano.it (M.B.); davide.perrotta@hotmail.it (D.P.); gloria.difilippo@unicusano.it (G.D.F.)

**Keywords:** sense of agency, tDCS, prefrontal cortex, intentional binding

## Abstract

**Objectives:** This research investigated the impact of transcranial Direct Current Stimulation (tDCS) on sense of agency (SoA) when focusing on cortical regions like the cerebellum, the dorsolateral prefrontal cortex (DLPFC), and the angular gyrus (AG). To this aim, three experiments were carried out, and agency was assessed through the Wundt Clock Paradigm, which provides a measure of intentional binding. **Methods:** The first experiment provided offline cathodal stimulation applied to the right cerebellum, with the return electrode placed on the left DLPFC, and participants were randomly assigned to either the placebo group or the active group. The second experiment adopted the same montage as the previous one, but the online stimulation was provided in a within-subjects design. **Results:** Since none of these studies targeting the cerebellum produced significant results on the agency measures, we carried out a third experiment aimed to replicate a previous study that provided inhibitory stimulation of the left AG. However, this also showed no modulations of SoA. **Conclusions:** Several explanations could be given for these negative results. For example, the inter-individual variability, task complexity, and limitations of tDCS technology may contribute to the inconsistencies of the results. Also, the failure to replicate a previous study raises the issue of the replicability crisis in psychology. Nevertheless, this study may represent an important reference for research aimed at modulating SoA through the neuromodulation of brain areas included in the agency network. Future studies could benefit from assessing individual cognitive abilities supporting agency, optimizing stimulation protocols, and exploring alternative brain stimulation techniques to obtain significant results.

## 1. General Introduction

Sense of agency refers to the personal perception of being the cause of one’s actions and their resulting effects. This intricate experience entails the belief that actions are voluntary, purposeful, and within one’s control. Serving as a pivotal element of human self-awareness, the sense of agency significantly influences our perception of autonomy, social responsibility, and the ability to make inferences about our performance or the consequences of our actions [1,2,3].

This concept has important implications for both neurocognitive research and legal matters. The conscious awareness that healthy adults have of their intentions, actions, and consequences is relevant to understanding concepts like liability. For example, individuals who do not experience agency of actions may not be considered legally responsible for their behavior [4,5,6,7,8].

Implicit components of agency, as outlined by Siebertz and Jansen [9], are typically evaluated using indirect methods like the Wundt Clock Paradigm and intentional binding, which measure the subjective perception of the temporal connection between one’s actions and their consequences [10,11,12,13,14,15]. Interestingly, several studies have investigated the effect of transcranial Direct Current Stimulation (tDCS) and Transcranial Magnetic Stimulation (TMS) on the sense of agency by targeting different cortical regions, including the dorsolateral prefrontal cortex (DLPFC), presupplementary motor areas (pre-SMA), temporoparietal junction (TPJ), and angular gyrus (AG) [16,17,18,19,20,21]. Notably, many empirical investigations employ excitatory stimulation, e.g., [17], while Cavazzana et al. [18] used offline tDCS to either inhibit or excite the primary auditory cortex (PAC), leading to a decrease in sense of agency (SoA). Marotta et al. [22] adopted offline stimulation in two experiments to target the premotor cortex and the cerebellum. The aim was to assess the subjects’ ability to recognize their own agency of movement by being able to observe only the posture of a rubber hand, whose movement (passive movement) might either align with or contradict the movement of the subject’s concealed hand (active movement). The findings showed that anodal stimulation of the premotor cortex diminished sense of agency in the active congruent state, while no effects were observed in any of the other conditions, including cerebellum stimulation. Furthermore, a meta-analysis by Khalighinejad et al. [17] compared seven experiments with online anodic stimulation of DLPFC, suggesting the crucial role of this cortical region in the sense of agency. All the experiments adopted the clock paradigm and, in most cases, anodic and sham stimulation of the left DLPFC. Two experiments documented an increase in agency after active stimulation.

## 2. Study 1

### 2.1. Introduction

Considering the well-established role of the prefrontal cortex (PFC) in agency but the inconsistent results of prior studies on PFC stimulation, we adopted a tDCS approach involving frontal disfacilitation through cerebellar inhibitory stimulation (see [23] for details on this approach). In fact, empirical observations suggested that this type of stimulation might exert an indirect impact on the prefrontal cortex, as demonstrated, for example, by cathodal cerebellar tDCS (ctDCS) effects on cognitive enhancement [24,25]. This aligns with the contralateral connectivity between the cerebellum and the cortex [26,27]. This proposal is further supported by studies that report consistent results with cathodal cerebellar stimulation and anodal stimulation of the DLPFC [28,29,30]. Also, in clinical studies utilizing tDCS, concurrent stimulation of the prefrontal cortex and cerebellum has been employed to engage the entire network [31,32], further supporting the overarching concept of complementary functioning between the PFC and the cerebellum.

As mentioned above, the PFC has been recognized as a key area in processing the SoA, but possible effects of tDCS stimulation of this area were suggested, even if not always consistent [17,33]. For these reasons, in the current study, cathodal stimulation was applied to the right cerebellum with the return electrode over the lDLPFC; the hypothesis was to enhance the sense of agency to a greater extent when compared with studies merely stimulating the DLPFC.

### 2.2. Materials and Methods

#### 2.2.1. Participants

Fifteen participants were selected at the University Niccolò Cusano (9 females, mean age 27.53 ± 8.76). Subjects were recruited from the psychology faculty at the university Niccolò Cusano. They had normal or corrected vision and were administered an anamnestic questionnaire to exclude neurological or psychological disorders. Also, they were tested for the tDCS eligibility before being enrolled in the experiment. Participants were randomly assigned to the active group (N = 7, mean age 28.5 ± 9.24) or sham group (N = 8, mean age 27.28 ± 8.98) in a between-subjects design. All subjects were right-handed [34].

#### 2.2.2. Task and Procedure

We used the Wundt Clock Paradigm as an implicit measure of agency [11]. All participants signed the informed consent and performed the Wundt Clock Paradigm twice during a session: once before and once after the offline tDCS stimulation, in a between-subjects design.

Subjects were asked to look at a round clock face (diameter = 8 cm), presented centrally on a computer screen and composed of 60 dots (diameter = 2 mm). In each trial, a red dot (diameter = 4 mm) rotates clockwise around the dots at a speed of 2560 milliseconds per rotation, beginning from a random clock position.

**Figure 1:**
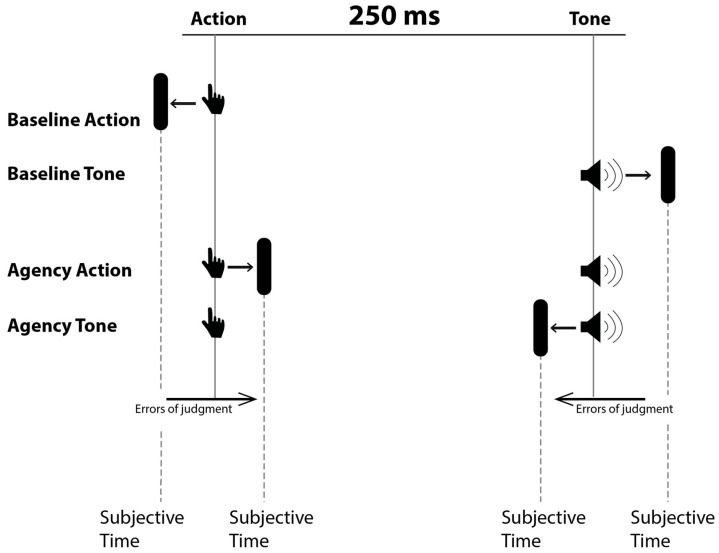
The four blocks of Wundt’s clock paradigm. From the top to the bottom, the first two blocks represent baseline measurements. The last two require subjective recognition of the time in which the action or tone occurred.

As shown in Figure 1, four different blocks were provided, which differed in terms of the event to be evaluated (action or tone) and the presence or absence of a relationship between the action and the tone. In the agency condition, participants were instructed to press the spacebar (random choice) on the keyboard with their dominant hand. After pressing the spacebar, a brief tone (frequency = 600 Hz, duration = 75 ms) was played with a 250 ms delay, while the red dot on the clock kept rotated (random duration from 1000 to 2000 ms in 250 ms intervals) before disappearing. When the red dot on the clock disappeared, participants were required to indicate the perceived position of the dot either at the button press (agency action) or at the tone onset (agency tone). In the two basic conditions, time judgements were made on the action (baseline action) or tone (baseline tone) occurring in isolation.

The four blocks were randomly administered. Each block included 15 trials, for a total of 60 trials. The task lasted about 15 min depending on the individual rest time and was repeated twice: before and after 18 min of tDCS stimulation. The whole experiment lasted about 50 min.

**Figure 2:**
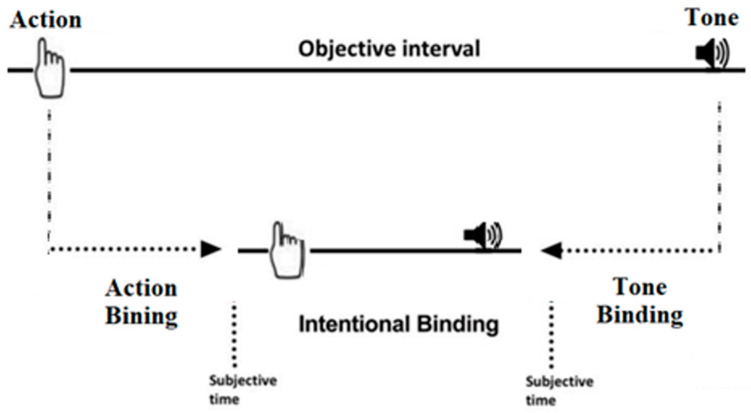
A visual representation of intentional binding. The upper section shows the objective timeline of the action and tone’s occurrence. The lower section displays the subjective time perception of the action and tone in the given task.

The Wundt Clock Paradigm provides three main measures (Figure 2): action binding, tone binding, and intentional binding, indicating the difference between the average judgment errors in the action or tone agency and the mean errors of judgement in the action or tone baseline, respectively. Intentional binding is calculated by subtracting the binding between the action and tone from the actual distance between them (250 ms).

#### 2.2.3. tDCS and Study Design

**Figure 3:**
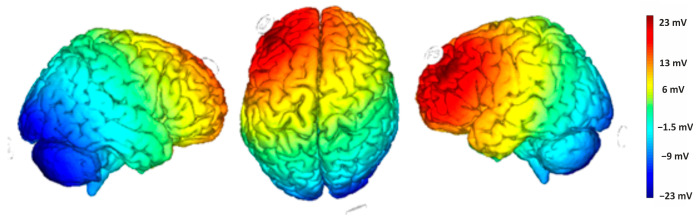
Electric field modeling for the right cerebellum/F3 tDCS montage. Simulation was performed with NIC 2 software.

Direct current was delivered through a pair of saline-soaked surface sponge electrodes (25 cm^2^) and administered by a battery-powered, software-controlled constant current stimulator (BrainSTIM 2.3, EMS srl, Bologna, Italy [35,36]). We employed a randomized, double-blind, sham-controlled protocol, with the target electrode delivering negative current (cathodal stimulation) to the right cerebellum (1 cm below and 4 cm to the right of the inion) and the return electrode placed on the left DLPFC (F3, based on the international EEG 10/20 system). The simulation of the cortical electric field distribution was carried out using the NIC 2 software (version 2.0.11), as depicted in Figure 3. For the active stimulation, the current intensity gradually increased over 10 s at the start of the session (ramp up), was delivered at −2.0 mA for 20 min, and then decreased over 10 s at the end of the session (ramp down) to reduce the sensation. In the sham stimulation, the ramp-up lasted 10 s until reaching −2.0 mA, followed by 7 s of current delivery and a 10 s ramp down. After 20 min without stimulation, the ramp up-ramp down cycle was repeated at the end of the session. Participants were asked to guess the assigned group (active or sham), but their guesses were at chance level, confirming the blind condition.

**Figure 4:**
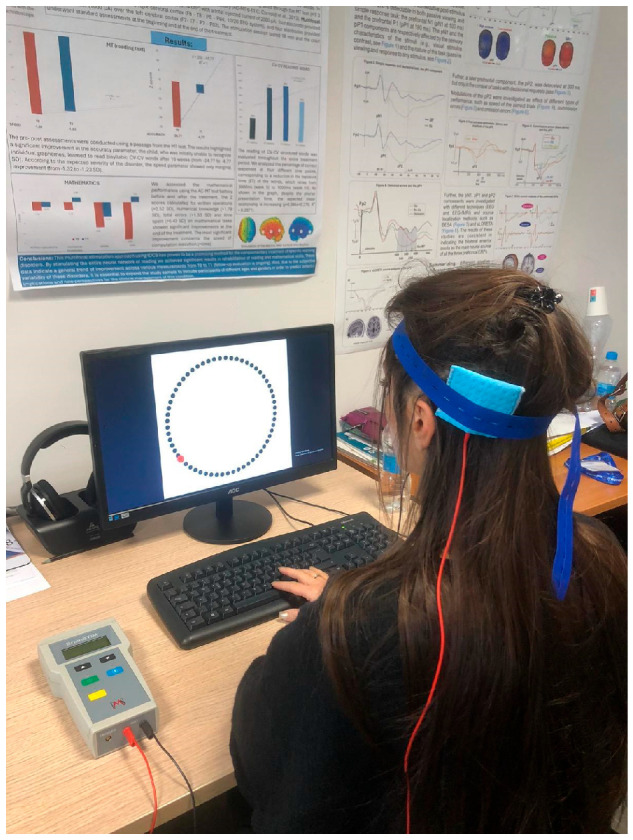
Experimental setting: the participant is performing the Wundt Clock Paradigm while receiving the tDCS stimulation.

Potential adverse effects of tDCS were evaluated by the experimenter at the end of each session through an interview based on the questionnaire by Brunoni et al. [37]. None of the participants reported any notable adverse effects. The experimental setting is shown in Figure 4.

### 2.3. Data Analysis

Statistical analysis was carried out using Statistica 10 software [38]. The considered parameters were Action Binding, Tone Binding, and Intentional Binding. With the aim of testing mean differences between active and sham stimulation, the mixed design ANOVA model was performed with group (Cathodal vs. Sham) as between-subjects factor and session (prestimulation vs. post-stimulation) as within-subjects factor. The overall α level was set at 0.05. Results were adjusted for multiple comparisons using Fisher’s Least Significant Difference (LSD) test, and effect size was calculated as partial eta squared ηp^2^, ≥0.01 for small effect, ≥0.06 for moderate effect, and ≥0.14 for large effect [39].

### 2.4. Results

No significant effects emerged from statistical analysis for the main factors or the interaction effects on Action Binding (F_1,13_ = 0.95, *p* = 0.35, ηp^2^ = 0.07), Tone Binding (F_1,13_ = 0.0, *p* = 0.96, ηp^2^ = 0.00), or Intentional Binding (F_1,13_ = 0.13, *p* = 0.73, ηp^2^ = 0.01).

### 2.5. Discussion

To the best of our knowledge, this was the first study in the field adopting a cerebellar–prefrontal tDCS montage. However, in contrast to the original hypothesis, the active stimulation did not produce significant effects on the considered measures of agency. A possible explanation for this result could be the use of the offline rather than online tDCS. In fact, the literature showed that timing of stimulation is not trivial and that it is hard to predict the differential effects of offline instead of online stimulation on the specific domains (see, e.g., [40]). Therefore, future investigations testing the alternative approach are needed before concluding that this montage is not effective in affecting agency.

## 3. Study 2

### 3.1. Introduction

Study 1 did not lead to the expected effects on agency. Investigating the reasons for this outcome, we hypothesized the need for online rather than offline stimulation.

Within the scope of research, many researchers are investigating the effectiveness of the online versus offline application of tDCS, and the findings were inconsistent depending on experimental designs, target population, cognitive tasks, or outcome measures (see [40] for a review). Overall, inconsistent results were mainly associated with brain-area- or task-related effects [24,41].

As for the specific domain of agency, both offline and online tDCS have been tested [16,17,18,22], but findings did not indicate greater benefits for either approach. However, since none of the previous studies targeted the cerebellum, the question relating to the effects of online cerebellar stimulation is still open.

In fact, following the rationale of Study 1, our hypothesis was that ctDCS concomitant to the agency task could allow a disfacilitation of DLPFC with relevant effects on the considered measures. Moreover, unlike Study 1, we adopted a within-subjects design to reduce any variability between groups.

### 3.2. Materials and Methods

#### 3.2.1. Participants

Fourteen subjects were gathered at the University Niccolò Cusano (7 females, mean age 23 ± 2.42). Subjects were recruited from the psychology faculty at the university Niccolò Cusano. They had normal or corrected vision and were administered an anamnestic questionnaire to exclude neurological or psychological disorders. Also, they were tested for tDCS eligibility before being enrolled in the experiment. All subjects were right-handed [34].

#### 3.2.2. Task and Procedure

All participants were enrolled in both active and sham stimulation in two different sessions with a random order (within-subjects design). The second session occurred between 2 and 7 days after the first one. After signing the informed consent, the tDCS was applied (see below) and the current delivered; after 5 min of stimulation, the Wundt Clock Paradigm (see Methods of Study 1 for details) was provided as well (online stimulation, i.e., the task was concomitant to the tDCS).

Depending on individual pauses, the task lasted approximately 5 min less or more than the stimulation, and the whole session lasted about 30 min. When the task finished earlier, the subject waited until the conclusion of the stimulation.

#### 3.2.3. tDCS and Study Design

Direct current was applied through a pair of surface sponge electrodes soaked in saline (25 cm^2^) and powered by a battery-operated constant current stimulator. We used a randomized, single-blind, sham-controlled protocol with the target electrode over the right cerebellum (1 cm below and 4 cm to the right of the inion) and the return electrode on the left DLPFC (F3 according to the international EEG 10/20 electrode placement) (see Figure 3). The target electrode delivered a negative current (cathodal stimulation) delivered by the BrainStim 2.3 software [36], which allows the experimenter to set the stimulation mode (cathodal or sham). For the active stimulation, the current intensity gradually increased over 10 s at the start of the session (ramp-up), was maintained at −2.0 mA for 20 min, and then gradually decreased over 10 s at the end of the session (ramp-down) to reduce its perception. During the sham stimulation, the ramp-up was applied for 10 s until reaching −2.0 mA, the current was administered for 7 s, and was followed by a 10 s ramp-down. After 20 min of no stimulation, the ramp-up and ramp-down cycle was repeated at the end of the session. Participants were asked to guess their assigned group (active or sham), but their guesses were at chance level confirming the blind condition.

At the end of each session, participants were administered an interview based on the Brunoni and colleagues’ questionnaire [37]; none of the participants reported any significant adverse effects.

### 3.3. Data Analysis

Statistical analyses were performed using Statistica software [38]. The considered parameters were Action Binding, Tone Binding, and Intentional Binding. With the aim of testing mean differences between active and sham stimulation, the repeated measures analysis of variance (ANOVA) model was performed with group (Sham–Cathodal vs. Cathodal–Sham) and Stimulation (Cathodal vs. Sham) as factors. The overall α level was set at 0.05. Results were adjusted for multiple comparisons using Fisher’s Least Significant Difference (LSD) test, and effect size was computed using partial eta squared (ηp^2^).

### 3.4. Results

No significant effects emerged from statistical analyses, neither for the main factors nor the interaction effects on Action Binding (F_1,12_ = 0.078, *p* = 0.784, ηp^2^ = 0.006), Tone Binding (F_1,12_ = 1.923, *p* = 0.191, ηp^2^ = 0.138), or Intentional Binding (F_1,12_ = 2.017, *p* = 0.181, ηp^2^ = 0.144).

### 3.5. Discussion

After obtaining non-significant results in Study 1, we hypothesized that the lack of expected results could be due to the use of offline tDCS, considering the conflicting results in the literature regarding the efficacy of online tDCS compared with offline tDCS [40]. In addition, we decided to adopt a within-subject design to control for individual variability [42,43]. Despite these methodological adjustments, Study 2 did not yield significant effects. This outcome suggests that online cerebellar–prefrontal tDCS, similar to the offline approach, does not significantly influence the sense of agency, likely due to the reduced effects of cerebellar tDCS on the DLPFC [23] or individual differences in responses to tDCS [44]. It is conceivable that genetic, anatomical, and neurophysiological differences may influence the results. Since these studies failed to suggest any effect of cerebellar tDCS on agency, further research is required to examine the role of prefrontal tDCS in modulating agency.

## 4. Study 3

### 4.1. Introduction

Given the absence of significant effects in the first two studies, we decided to carry out a replication study by referring to the tDCS experiment by Khalighinejad and Haggard [16], who achieved significant effects on agency by stimulating the left angular gyrus (AG). The AG has been recognized as a critical region for explicit judgments of agency, as shown by neuroimaging studies that have detected activation of the AG in agency processing [45,46,47,48], and both lesion [49] and neurostimulation [50] studies underlined its key role in intentional action and agency. In particular, the AG is responsible for processing disparities between intended action and its actual consequences [46], and it is active when sense of agency is compromised [47,51,52]. As a further confirmation, dysfunctions of the superior parietal lobe, either caused by lesions [53] or tDCS [16], have been linked to impaired sensorimotor integration and disrupted sense of agency. Brain imaging studies have shown engagement of the angular gyrus in routine low-agency tasks in which subjective feedback was not related to one’s action (for a meta-analysis, see [48]). For example, the study by Chambon et al. [54] showed larger recruitment of the angular gyrus when participants’ evaluations of agency were low rather than when evaluations reflected higher agency. Furthermore, Chambon and colleagues [55] also demonstrated that the fluidity of action selection processes provides insight into the sense of agency. Specifically, the AG computes the subjective sense of control over the outcomes of an action before the action is actually performed. It is important that this calculation arose before any (retrospective) comparison between the action being prepared and its expected outcomes. Monitoring these signals in the AG could serve as a reliable indicator of intent, helping to prevent agency illusions that may arise from relying on post hoc judgments of the action’s effect associations. Specifically, the monitoring of internal action selection processes can make the sensation of being in control when combined with the monitoring of external action outcomes. The study suggested that the AG tracks signals associated with action selection to proactively guide subjective judgments of control over action outcomes [54], while Van Kemenade and colleagues [56] underlined the AG role for a supramodal comparison in action–outcome monitoring.

Khalighinejad and Haggard [16] described three experiments of tDCS stimulation of the AG: in Experiment 1, anodal stimulation of the left AG produced a significant decrease in the binding between action and outcome, with an anticipation of tone and consequently a decrease in the time link between action and outcome (intentional binding), which resulted in a decrease in agency. Afterward, the authors attempted to identify whether there might be a difference between anodal and cathodal stimulation of AG (Experiment 2), assuming that if anodal stimulation of AG had decreased SoA, then cathodal stimulation should increase SoA. This experiment, however, did not lead to the expected results. Moreover, in a third experiment, the authors investigated hemispheric specialization and replicated the effects of left anodal AG stimulation for right- and left-hand actions, while no effects of right anodal AG stimulation were observed.

According to this, in the present study, we aimed to replicate Experiment 1 by Khalighinejad and Haggard [16], expecting to observe a decrease in agency as an effect of the left angular gyrus stimulation.

### 4.2. Materials and Methods

#### 4.2.1. Participants

Fifteen volunteers were gathered at the University Niccolò Cusano (8 females, mean age 24.87 ± 7.99). All participants were involved in active and sham stimulation. Subjects were recruited from the psychology faculty at the university Niccolò Cusano. They had normal or corrected vision and were administered an anamnestic questionnaire to exclude neurological or psychological disorders. Also, they were tested for tDCS eligibility before being enrolled in the experiment. All subjects were right-handed [34].

#### 4.2.2. Task and Procedure

All participants underwent both active and sham stimulation in two separate sessions at a random order (within-subjects design). The second session occurred between 2 and 7 days after the first. Each session lasted 30 min. Wundt Clock Paradigm was administered as a hidden measure of sense of agency (see Methods of Study 1 for details). After signing the informed consent, the tDCS was mounted (see below) and the current delivered; after 5 min of stimulation, the Wundt Clock was provided as well (online stimulation). Depending on individual pauses, the task could end approximately 5 min before or after the stimulation. If the task finished earlier, the subject waited until the end of the stimulation.

#### 4.2.3. tDCS and Study Design

**Figure 5:**
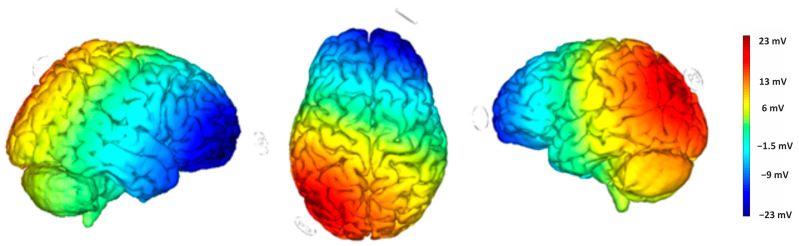
Electric field modeling for the P3/right supraorbital tDCS montage. Simulation was performed with NIC2 software.

Direct current was applied through a pair of surface sponge electrodes soaked in saline (25 cm^2^) and powered by a battery-operated constant current stimulator. We used a randomized, single-blind, sham-controlled protocol with the target electrode on the left angular gyrus (P3 according to the international EEG 10/20 electrode placement) and the return electrode over the right supraorbital area, above the left eyebrow. The target electrode provided a positive current (anodic stimulation) delivered by the BrainStim 2.3 software [36]. The simulation of the cortical electric field distribution was carried out using the NIC 2 software, as depicted in Figure 5. For the active stimulation, the current intensity gradually rose over 10 s at the start of the session (ramp-up), was maintained at 2.0 mA for 20 min, and then decreased over 10 s at the end of the session (ramp-down) to reduce its perceptibility. In the sham stimulation, the ramp-up was applied for 10 s until it reached 2.0 mA, followed by 7 s of current delivery and a 10 s ramp-down. After 20 min of no stimulation, the ramp up-ramp down cycle was repeated at the end of the session. Participants were asked to guess their assigned group (active or sham), but their guesses were at chance level, confirming the blind condition.

Potential adverse effects of tDCS were evaluated by the experimenter at the end of each session through an interview based on the questionnaire by Brunoni et al. [37]; none of the participants reported any significant adverse effects.

### 4.3. Data Analysis

Statistical analyses were performed using the Statistica software [38]. The considered parameters were Action Binding, Tone Binding, and Intentional Binding. With the aim of testing mean differences between active and sham stimulation, the repeated measures analysis of variance (ANOVA) model was performed with group (Sham–Anodic vs. Anodic–Sham) and Stimulation (Anodic vs. Sham) as factors. The overall α level was fixed at 0.05. Results were adjusted for multiple comparisons using the Fisher’s Least Significant Difference (LSD) test, and effect size was computed using partial eta squared (ηp^2^).

### 4.4. Results

No significant effects emerged from statistical analysis for the main factors or the interaction effects on Action Binding (F_1,13_ = 2.202, *p* = 0.162, ηp^2^ = 0.145), Tone Binding (F_1,13_ = 0.006, *p* = 0.941, ηp^2^ = 0.000), or Intentional Binding (F_1,13_ = 1.14 *p* = 0.305, ηp^2^ = 0.081).

### 4.5. Discussion

Despite attempting to replicate the experiment by Khalighinejad and Haggard [16] using identical methods, timing, stimulation, and tasks, our current study did not yield a significant difference between anodic and sham stimulation. While our findings did not support those of the initial experiment conducted by Khalighinejad and Haggard [16], they do align the results of their second experiment, which also highlighted the inconsistency of effects.

Although the results of this study are not statistically significant, we believe the information they provide is relevant, as they bring to light critical issues. In fact, it would seem that stimulation of the left AG is not such an effective strategy for modulating action. As a further point, the failure to replicate a previous study can be ascribed to several factors, such as the well-documented variability in tDCS effects on healthy individuals (for a review, see [44]), the physiological difficulty in modulating cognitive performance in healthy subjects [44,57], or the presence of interindividual variability for tDCS interventions [43].

## 5. General Discussion

In the three studies outlined above, we intended to explore the potential impacts of tDCS on implicit measures of sense of agency through various experimental approaches. In the first experiment, the offline cerebellar inhibitory stimulation was provided to induce a disfacilitation of the prefrontal cortex (for a review, see [23]), whose contribution in agency has already been documented (e.g., [17]). Despite our efforts, this approach did not yield significant effects. Then, a second experiment was carried out using the same methods but adopting online stimulation in a within-subjects design. In fact, the timing of the stimulation is still a subject of debate in the literature as it seems that sometimes providing the task during the stimulation can lead to better results than the offline approach in different domains (see, e.g., [40]), including agency (see [17]). However, experiment 2 did not produce significant results either. For this reason, we decided to carry out a third experiment with the aim to replicate the study by Khalighinejad and Haggard [16], who described modulations of agency by stimulating the angular gyrus (AG), already identified as a key area for explicit judgments of action [45,46,47,48]. However, the latter experiment failed to replicate the original findings. The lack of a positive result cannot be considered as a sufficient condition to contest the validity of the Khalighinejad and Haggard study. On the contrary, it allows the discussion to deepen by broadening the focus on different factors. For example, it could be hypothesized that negative results are due to hardware failure or an inappropriate experimental setup. However, we tend to exclude this in our study, as the tDCS montage and impedance check were carried out by expert operators, and the software would stop stimulation if current delivery or electrode placement changed. Also, the device was powered by battery and not by electricity to prevent abnormal current peaks. Participants were regularly asked about the sensations (e.g., tingling) associated with the stimulation to further confirm the regularity of the procedure. A limitation could be the absence of other sources of supplementary evidence or secondary outcomes (e.g., other mental chronometry measures). In fact, future studies need to be carried out to draw more solid conclusions. It is, however, noteworthy that the authors of the original study [16] also provided the same montage in two different experiments, obtaining contrasting results.

The reasons for the inconsistent findings may be attributed to many factors, such as the well-known difficulties of tDCS in modulating cognitive abilities in healthy subjects [44,57]. Therefore, considering that −2 mA was used as a power intensity in these studies, an attempt could be made in future studies to increase the current intensity (e.g., to 2.5–3.0 mA), which could produce more robust effects. More broadly, this study also calls into question the hot topic of the replicability crisis in psychology [58,59,60]. As for the use of tDCS in healthy samples, it should be noted that stimulation effects can be attributed to stable factors, such as genetic, morphological, and demographic peculiarities, or to variable factors, such as participants’ alertness and changes in hormonal activity (see [43]), as well as to contextual elements, commitment, or the participants’ skills needed to perform the agency tasks of attention, visuospatial memory, and visual abilities, [61]. As for the crisis of replicability in psychology, it refers to the difficulty of obtaining the same findings as previous studies and theories. This is due to multiple factors, including the characteristics and number of subjects used for the studies [60], as well as the reference theories of the studies [59]. In fact, it has been shown that out of 100 experimental studies published in three different psychology journals, only 36% of them have been shown to be replicable [62]. Another issue to consider is statistical power, which was found to be low for some analyses of the present experiments. This suggests further caution in interpreting the results, and investigations with larger sample sizes and statistical power are needed to draw more accurate conclusions.

Future studies on this topic could also use other devices such as ultrasound stimulation or multifocal tDCS to stimulate the agency networks (e.g., prefrontal, premotor area, and angular gyrus). Furthermore, in addition to the targeted areas, the contribution of other regions such as the primary motor cortex and the premotor cortex needs to be considered as well when investigating SoA. Indeed, these motor areas play a crucial role in the generation of action, contributing to the process of predicting motor consequences [18,21,28,51]. For example, by stimulating the M1, Marotta et al. [21] highlighted changes in the subjective experience of agency, while Cavazzana et al. [18] anodically stimulated the premotor cortex, reporting a reduction in SoA, as the thalamus plays a crucial role in the temporal synchronization between intention, action, and perception of effects, fundamental elements for SoA [28,51].

In addition, given the difficulty of neuromodulating cognitive abilities in healthy subjects, it may be worthwhile to extend the studies by investigating similar methods in clinical populations. Also, different measures of agency could be considered as explicit and implicit tests (see [61] for possible limitations of the Wundt Clock Paradigm), and the relationship between SoA and other cognitive domains should be deepened. In fact, Hon [63] suggested a relationship between the sense of agency and the attention needed to process agency, also indicating SoA as a conscious cognitive process influenced by working memory and attention. Replication studies play a pivotal role in validating research outcomes and resolving inconsistencies, thereby enhancing the reliability and robustness of scientific knowledge in this area.

## 6. Conclusions

The present studies illustrate the challenges in using transcranial Direct Current Stimulation (tDCS) to modulate the sense of agency. While no significant effects were observed, these findings highlight the complexities of individual variability, methodological constraints, and the limitations of tDCS in healthy populations. This work also contributes to ongoing debates about the replicability crisis and methodological rigor in psychology. Indeed, these results emphasize the importance of replicating prior studies and exploring alternative neuromodulation techniques. Future research should focus on refining experimental designs, targeting clinical populations, and investigating the interplay between agency and cognitive functions.

## Data Availability

The data supporting the conclusions of this article will be made available by the authors on request. The data are not publicly available due to privacy.
